# Time spent in a better cardiovascular health and risk of cardiovascular diseases and mortality: a prospective cohort study

**DOI:** 10.1186/s12967-023-04252-x

**Published:** 2023-07-14

**Authors:** Qiuyue Tian, Shuohua Chen, Xiaoni Meng, Haotian Wang, Cancan Li, Deqiang Zheng, Lijuan Wu, Aitian Wang, Shouling Wu, Youxin Wang

**Affiliations:** 1 Beijing Key Laboratory of Clinical Epidemiology, School of Public Health, Capital Medical University, 10 YouanmenXitoutiao, Beijing, 100069 China; 2 Department of Cardiology, Kailuan General Hospital, North China University of Science and Technology, 57 Xinhua East Road, Tangshan, 063000 China; 3 Department of Intensive Medicine, Kailuan General Hospital, Tangshan, 063000 China

**Keywords:** Cardiovascular health, Duration, Cardiovascular diseases, All-cause mortality

## Abstract

**Background:**

The protective effect of a higher ideal cardiovascular health (CVH) score on cardiovascular diseases (CVDs) and mortality is well recognized. However, little is known regarding the length of favorable CVH status associated with CVDs and mortality. This study aimed to examined whether the duration of better (ideal or intermediate) CVH is associated with risk of developing CVDs and mortality.

**Methods:**

This prospective cohort study used data from 83,536 individuals from 2006 to 2020 who were enrolled in the Kailuan Study. The CVH scores of individuals were assessed at visits 1, 2, 3, and 4, respectively. The years spent in better CVH were estimated for each individual as the number of examination cycles (0–4) in which the participant was in that CVH score ≥ 8 multiplied by 2 (the mean year interval of each visit). The primary outcomes are CVD events and all-cause mortality.

**Results:**

After a median follow-up period of 7.48 years, 5486 (7.07%) cases of incident CVD events and 7669 (9.18%) deaths occurred. Compared with participants in “ ≤ 4 years” group, those who maintained for > 4 years had less likely to develop adverse outcomes (CVD events: hazard ratio (HR): 0.60, 95% confidence interval (CI 0.56–0.63; all-cause mortality: HR: 0.77, 95% CI 0.74–0.81). The number of years spent in better CVH was nonlinearly correlated with CVD events or mortality (all *Ps* for nonlinear < 0.05). The results indicated that maintaining more than 6 years in a better CVH status was associated with a decreased risk of CVD events or mortality.

**Conclusion:**

Our study indicates that individuals maintaining more than 6 years in better CVH could increase cardiometabolic benefits and a lower risk of all-cause mortality.

**Supplementary Information:**

The online version contains supplementary material available at 10.1186/s12967-023-04252-x.

## Background

Globally, it is estimated that 17.9 million (32%) people died from cardiovascular diseases (CVDs) in 2019, and 85% of those deaths were due to heart attack and stroke [1]. In China, the prevalence of CVDs is rising, and the number was up to 330 million people in 2018 [2]. In 2010, the American Heart Association developed a new concept, cardiovascular health (CVH), which is defined by health behaviors (smoking, body mass index (BMI), physical activity, and diet) and health factors (total cholesterol (TC), blood pressure (BP), and fasting blood glucose (FBG)), focusing on prevention and reducing the burden of CVDs [3]. Previous studies showed that ideal CVH metrics or higher CVH scores were associated with a decreased risk of CVDs, stroke, heart failure (HF), and mortality [4–12], which presented an inverse gradient curve [13]. Data from the Kuopio Ischemic Heart Disease study indicate that ideal CVH metrics were inversely associated with acute myocardial infarction (MI), sudden cardiac death, and all-cause mortality among Finnish men during a follow-up period of 25 years [4, 14]. These studies assessed CVH status using a single point and did not consider changes in CVH over time.

Recently, studies examined the association between CVH score trajectories or CVH metrics change over time and CVDs or mortality during follow-up [15–17]. Previous studies indicated that participants with the high-stable II trajectory of CVH score had 76–79% decreased risk of CVDs and mortality compared with the low-stable group [16, 18]. These findings suggest the importance of maintaining a higher CVH score or ideal CVH metrics for preventing CVDs. However, few studies have considered the relationship between the duration spent in a better CVH category and health outcomes. Only two prospective cohort studies from the Framingham Offspring Study and the Korean Genome and Epidemiology Study Ansung-Ansan cohort study investigated the association between the duration of maintaining a better CVH and clinical outcomes [19, 20]. Although these above studies quantified the duration spent in a specific CVH category, it is not considered the dose–response relationship of time spent in a better CVH status with health outcomes.

Up to now, no prospective cohort study was performed to explore the association of the duration maintaining a better CVH with cardiovascular outcomes and all-cause mortality in China. Therefore, in this study, we examined whether the duration of a better CVH category is associated with the incidence of CVD events and their subtypes or mortality. Additionally, we explored the dose–response relationship of time spent in a better CVH status with health outcomes. To the above aim, we analyzed data from the Kailuan Study, a community-based prospective cohort in China.

## Methods

### Study sample

The Kailuan Study is a prospective cohort in the Kailuan community in Tangshan, Hebei, China. From 2006 to 2007, 101,510 adults (81,110 men and 20,400 women) aged 18 to 98 years were enrolled at baseline and completed routine medical examinations every 2 years at the Kailuan community by face-to-face interviews [21–23]. The objective and design have been described previously and registered in the Chinese Clinical Trial Registry (Registration number: ChiCTR-TNRC-11001489). For the present study, participants of the Kailuan study who attended examination visits 1 (2006–2007), 2 (2008–2009), 3 (2010–2011), and 4 (2012–2013) were eligible. We excluded participants who had any of the following conditions at these examinations (visit 1-visit 4): BMI less than 18.5 kg/m^2^, missing CVH components, died, and occurring CVD events. Data from examination visits 1, 2, 3, and 4 were included to calculate the duration of maintaining in better CVH score category. This study was approved by the ethics committees of Kailuan General Hospital (Approve No.: 2006–5). A written informed consent form was obtained from all participants.

### Definition of CVH score

According to the American Heart Association developed CVH score, CVH status was described by smoking, diet, physical activity, BMI, BP, FBG, and TC [3]. As the information on diet was not included in the questionnaire used in the Kailuan study, salt preference was considered a surrogate marker [24, 25]. Salt preference was divided into “low”, “medium”, and “high” based on the questionnaire. The detailed information is described in the Additional file 1: Table S1. The total score range was 0 to 14 [26]. Scores of 0–7, 8–11, or 12–14 points were regarded as having poor, intermediate, or ideal CVH, respectively [19, 20].

Because of the relatively low number of participants with ideal CVH status, we combined the intermediate and ideal CVH status. The number of years lived in a better (intermediate or ideal) CVH status was estimated for each individual as the number of examination cycles (0–4) in which the participant was in that CVH score ≥ 8 category (intermediate or ideal) multiplied by 2 (the mean year interval of each visit). For example, if an individual scored 14 (ideal) at visit 1, scored 13 (ideal) at visit 2, scored 10 (intermediate) at visit 3, and scored 7 (poor) at visit 4 (8 years from visit 1), he/she would hypothetically maintain intermediate or ideal CVH for 6 years since the visit 1. In addition, participants were further divided into two groups (“ ≤ 4 years” or “ > 4 years”) based on the median years spent in better CVH status.

### Covariates

Standardized questionnaires were used to collect sociodemographic characteristics [21–23], lifestyle factors, and medical history, including age, sex, types of work, seat time, educational level, drinking status, family per-member monthly income, and history of diseases (hypertension, diabetes, and hyperlipidemia). These covariates were updated at every follow-up (in 2014, 2016, and 2018).

### Follow-up and outcomes

All participants were followed up every 2 years until death or December 31, 2020. The primary outcomes were the occurrence of the following events during follow-up after visit 4: first incident CVD events (including MI, stroke, atrial fibrillation (AF), and HF) and all-cause mortality. Information on diagnoses of CVD events was obtained from medical records from medical insurance or hospitals [22]. Information on diagnoses of death was obtained from family reports, death certificates from provincial vital statistics offices, and medical records from medical insurance or hospitals [22]. The secondary outcomes were the occurrence of the CVD subtypes, including MI, AF, stroke, and HF.

### Statistical analysis

Baseline characteristics of participants were described by number (percentage) or median with interquartile range (IQR) and compared by using chi-square tests for the categorical variables and Kruskal‒Wallis test for the continuous variables.

Person-years were calculated from the date of visit 5 (2014–2015) to the date of death or the end of follow-up (December 31, 2020), whichever came first. The adjusted cumulative incidence of CVD events or all-cause mortality was estimated using the Kaplan‒Meier method and compared by log rank test. The proportional hazards assumption was tested by the Schoenfeld residuals, and no violation was found. The association of each better CVH duration category with the risk of CVD events and all-cause mortality was estimated using Cox proportional hazards regression models. The Cox model with restricted cubic splines (RCS) was used to flexibly model potential nonlinear associations between the numbers of years spent in a better CVH (0, 2, 4, 6, and 8 years) as a continuous variable and outcomes. All models were adjusted for age, sex, types of work, seat time, educational level, drinking status, family per-member monthly income, and history of diseases (hypertension, diabetes, and hyperlipidemia).

Sensitivity analyses were performed for the primary outcomes, with additional adjustments for CVH scores (visit 4). Considering non-CVD death as a competing risk event rather than a censoring event, a Fine-Gray competing risk model was applied to address this issue. Considering the time dependence of covariates, time-dependent Cox proportional hazards models were constructed while simultaneously adjusting for time-varying confounders and other covariates. We assessed whether age or sex modified the associations between the duration in a better CVH category and the risk of outcomes. A likelihood ratio test was conducted to examine statistical interactions by comparing -2 log-likelihood chi-square between nested models, with or without the multiplication interaction terms.

All statistical analyses were conducted using SAS, version 9.4 (SAS Institute Inc.). Two-sided *P* < 0.05 was considered statistically significant.

## Results

### Characteristics of participants

**Fig. 1:**
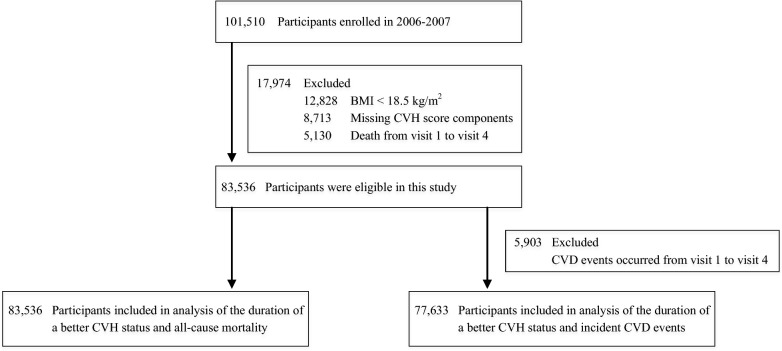
Derivation of Study Samples. *BMI* body mass index; *CVD* cardiovascular disease; *CVH* cardiovascular health

Of the 101510 subjects, we excluded 12828 with less than 18.5 kg/m^2^, 8713 with missing CVH components, and 5130 died from visits 1 to 4 (2006–2013). A total of 83536 subjects participated in the study. Additional exclusion of those occurring CVD events from visits 1 to 4 reduced the sample size to 77633 for analyzing the duration of CVH status and CVD events (Fig. 1).

The study included 83536 participants (median age 62 years, 19.79% female), of whom, 77633 were without CVD events at baseline (visit 4). Overall, 30150 (36.09%) had 4 or fewer years in a better CVH status and 53,386 (63.90%) had more than 4 years in a better CVH (Table 1). Compared with participants with shorter a better CVH duration (“ ≤ 4 years” group), those who maintained a better CVH for > 4 years were more likely to be younger, female, non-smokers, non-drinkers, to attain high school degree, to do active physical activity and to have a low-salt preference and less likely to have longer sedentary time and have a high proportion of hypertension, diabetes, hyperlipidemia, and metabolic abnormalities.

**Table 1:** Characteristics of participants at baseline (Visit 4) based on duration of time lived in intermediate or ideal CVH

Characteristics	Duration of intermediate or ideal CVH category, No. (%) of participants		*P*
	≤ 4 y (n = 30150)	> 4 y (n = 53386)	
Age, year	61.94 (13.07)	61.33 (15.82)	< 0.0001
Male	27647 (91.70)	39400 (73.80)	< 0.0001
BMI, kg/m^2^	25.76 (3.57)	23.92 (3.55)	< 0.0001
Physical labour	26847 (89.37)	45525 (85.61)	< 0.0001
Senior high school or above	5276 (18.08)	11440 (22.04)	< 0.0001
Sedentary time ≥ 8 h	1177 (3.90)	1961 (3.67)	< 0.0001
Income > 1000^a^	16479 (54.83)	26033 (48.90)	< 0.0001
Current smoking	14507 (48.12)	10443 (19.56)	< 0.0001
Current drinking	12688 (42.09)	12182 (22.82)	< 0.0001
Salt > 12 g/d	4262 (14.14)	2849 (5.34)	< 0.0001
Physical activity, active	3647 (12.10)	7523 (14.09)	< 0.0001
Hypertension	7650 (25.38)	4800 (8.99)	< 0.0001
Diabetes	2778 (9.21)	1011 (1.89)	< 0.0001
Hyperlipidemia	2453 (8.14)	1638 (3.07)	< 0.0001
SBP, mmHg	140 (22)	125 (24)	< 0.0001
DBP, mmHg	90 (16)	80 (12.33)	< 0.0001
WHR	0.91 (0.06)	0.90 (0.08)	< 0.0001
TG, mmol/L	1.51 (1.22)	1.13 (0.80)	< 0.0001
TC, mmol/L	5.36 (1.41)	4.80 (1.16)	< 0.0001
LDL-C, mmol/L	2.67 (1.19)	2.44 (0.98)	< 0.0001
HDL-C, mmol/L	1.36 (0.50)	1.40 (0.46)	< 0.0001
FBG, mmol/L	5.72 (1.58)	5.17 (0.86)	< 0.0001

Continuous variable was represented by median [(interquartile range (IQR)]. Qualitative variable was represented by number (%)

^a^Family per-member income > 1000Yuan/month

*BMI* body mass index; *CVH* cardiovascular health; *DBP* diastolic blood pressure; *FBG* fasting blood glucose; *HDL-C* high-density lipoprotein cholesterol; *LDL-C* low-density lipoprotein cholesterol; *SBP* systolic blood pressure; *TC* total cholesterol; *TG* triglycerides; *WHR* waist hip rate

### Duration of a better CVH and CVD events

**Fig. 2:**
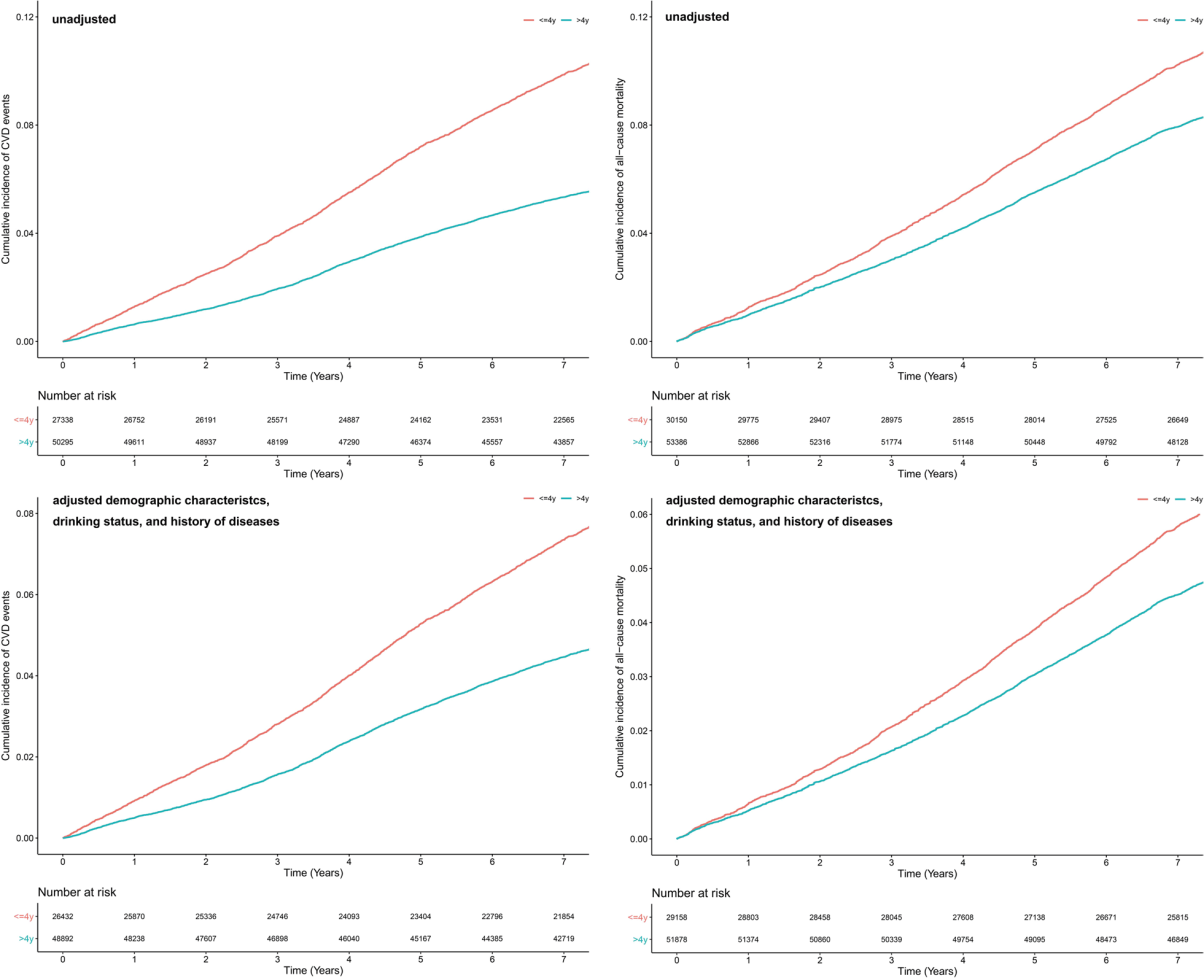
The unadjusted and adjusted cumulative incidence of CVD events and all-cause mortality. Based on the duration of intermediate or ideal CVH category, participants were divided into two groups (“ ≤ 4 years” or “ > 4 years”). *CVD* cardiovascular disease; *CVH* cardiovascular health

After a median follow-up period of 7.48 years (IQR: 7.00–7.86 years), 5486 (7.07%) incident CVD events and 7669 (9.18%) deaths occurred. Figure 2 shows the unadjusted and adjusted cumulative incidence of CVD events and all-cause mortality. The adjusted cumulative incidence of CVD events (7.22 ‰) and all-cause mortality (10.79 ‰) was the highest in the “ ≤ 4 years” group.

**Fig. 3:**
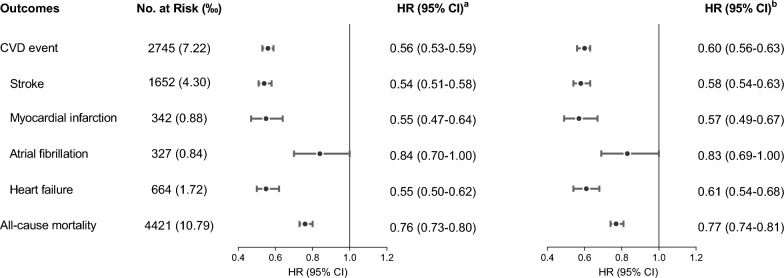
Forest plot for the association between maintaining intermediate or ideal CVH for above 4 years and the risk of outcomes. ^a^Model was adjusted for age and sex. ^b^Model was further adjusted for type of work, seat time, educational level, drinking status, family per-member monthly income, and history of diseases (hypertension, diabetes, and hyperlipidemia). *CI* confidence interval; *CVD* cardiovascular disease; *CVH* cardiovascular health; *HR* hazard ratio

Compared with individuals in the “ ≤ 4 years” group, the age- and sex-adjusted hazard ratio (aHR) were 0.56 (95% confidence interval (CI 0.53–0.59) for CVD events, 0.55 (95% CI 0.47–0.64) for MI, 0.54 (95% CI 0.51–0.58) for stroke, 0.84 (95% CI 0.70–1.00) for AF, and 0.55 (95% CI 0.50–0.62) for HF in individuals in the “ > 4 years” group (Fig. 3). In fully adjusted model, “ > 4 years” group was associated with lower CVD events (aHR: 0.60; 95% CI 0.56–0.63), MI (aHR: 0.57; 95% CI 0.49–0.67), stroke (aHR: 0.58; 95% CI 0.54–0.63), and HF (aHR: 0.61; 95% CI 0.54–0.68) in reference to ≤ 4 years of maintenance. However, participants who lived with > 4 years of a better CVH had lower AF risk (aHR: 0.83; 95% CI 0.69–1.00) yet without statistical significance.

**Fig. 4:**
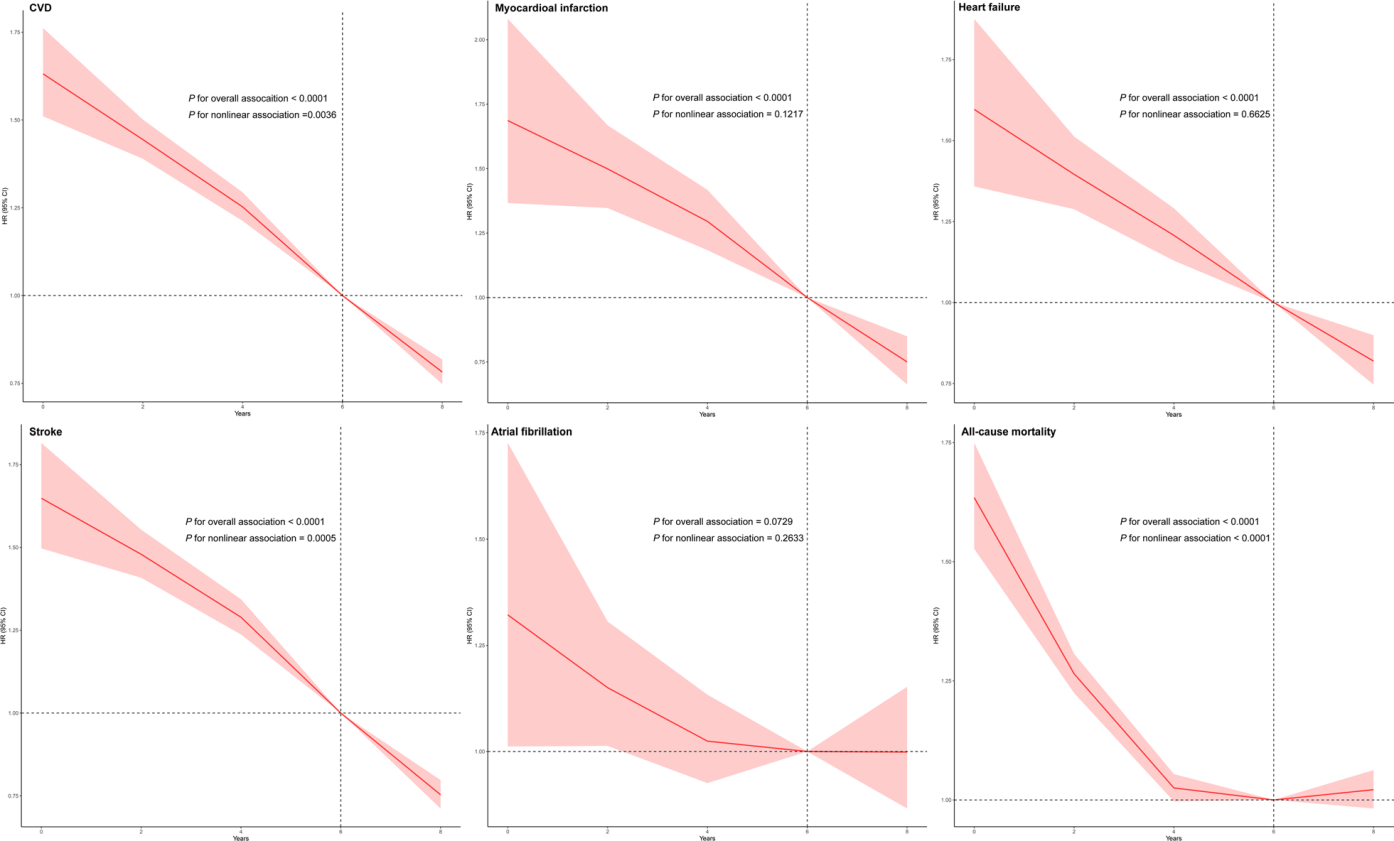
The nonlinear association of the number of years spent in a specific CVH with the risk of developing outcomes on follow-up. The restricted cubic splines model was used to flexibly model potential nonlinear association between the numbers of years spent in a specific CVH (0, 2, 4, 6, and 8) as a continuous variable and outcomes. The model was adjusted for age, sex, type of work, seat time, educational level, drinking status, family per-member monthly income, and history of diseases (hypertension, diabetes, and hyperlipidemia) *CI* confidence interval; *CVD* cardiovascular disease; *CVH* cardiovascular health; *HR* hazard ratio

The association between the number of years spent in a better CVH (0, 2, 4, 6, and 8) on a continuous scale and the risk of CVD events or their subtypes was declining curve shaped; living in a better CVH for > 6 years was associated with significantly lower risk for CVD events and their subtypes (*P* for overall association < 0.0001), except for AF (*P* for overall association = 0.0729) (Fig. 4). The association between the number of years spent in a better CVH and the risk of AF was flat-L shaped yet without statistical significance (Fig. 4). In addition, the number of years spent in a better CVH was nonlinear in the prognosis curves of CVD events and stroke (*P* for nonlinear < 0.05).

### Duration of a better CVH and all-cause mortality

Compared with the “ ≤ 4 years” group, participants who lived with > 4 years of a better CVH had lower all-cause mortality risk (aHR: 0.76; 95% CI 0.73–0.80) adjusting age and sex (Fig. 3). Similarly, longer ideal CVH duration was associated with a significantly lower risk for all-cause mortality (aHR: 0.77, 95% CI 0.74–0.81), further adjusting types of work, seat time, educational level, drinking status, family per-member monthly income, and history of diseases.

The number of years spent in a better CVH was nonlinear in the prognosis curves of mortality (Fig. 4, *P* for nonlinear < 0.05). The association between the number of years spent in a better CVH and the risk of all-cause mortality was U-shaped (Fig. 4). The number of years spent in a better CVH associated with the lowest risk of all-cause mortality was 6 years, although the associations were not statistically significant.

### Sensitivity and stratified analyses

After further adjusting the model for CVH score (visit 4) or time-dependent covariates, maintaining a better CVH for > 4 years was consistently associated with lower risks for CVD events, MI, stroke, HF, and all-cause mortality compared with ≤ 4 years of maintenance (Additional file 2: Table S2). In addition, considering non-CVD death as a competing risk event, these results were still similar.

There was no effect modification by sex of the associations of duration in a better CVH category with CVD events and dying (Table 2, *P* for interaction > 0.05). Nevertheless, the associations between the number of years in a better CVH category and CVD events or their subtypes were most pronounced in individuals aged less than 65 years (*P* for interaction < 0.05), except for AF. In addition, we found that the association varied in certain populations, and the effect was significantly modified by age, which was most pronounced in individuals aged less than 65 years (*P* for interaction < 0.05).

**Table 2:** Subgroups analyses of association between duration of time lived in intermediate or ideal CVH and outcomes

	CVD events	Stroke	MI	AF	HF	All-cause mortality
All participants	0.60 (0.56–0.63)	0.58 (0.54–0.63)	0.57 (0.49–0.67)	0.83 (0.69–1.00)	0.61 (0.54–0.68)	0.77 (0.74–0.81)
Sex						
Male	0.60 (0.57–0.64)	0.59 (0.54–0.63)	0.56 (0.48–0.67)	0.85 (0.70–1.03)	0.63 (0.55–0.71)	0.78 (0.74–0.82)
Female	0.61 (0.50–0.73)	0.59 (0.46–0.76)	0.83 (0.45–1.55)	0.78 (0.45–1.36)	0.52 (0.38–0.72)	0.71 (0.59–0.85)
*P* for interaction	0.3257	0.7200	0.8336	0.5827	0.0974	0.1136
Age						
Age < 65 years	0.54 (0.50–0.59)	0.53 (0.48–0.59)	0.51 (0.40–0.64)	0.81 (0.59–1.10)	0.52 (0.42–0.64)	0.67 (0.60–0.73)
Age ≥ 65 years	0.68 (0.63–0.73)	0.66 (0.59–0.73)	0.66 (0.53–0.82)	0.92 (0.73–1.16)	0.70 (0.61–0.82)	0.91 (0.86–0.96)
*P* for interaction	< 0.0001	< 0.0001	0.0365	0.3241	0.0003	< 0.0001

*P* for interaction was estimated by likelihood ratio test. The multivariable model was adjusted for age, sex, type of work, seat time, educational level, drinking status, family per-member monthly income, and history of diseases (hypertension, diabetes, and hyperlipidemia)

*AF* atrial fibrillation; *CVD* cardiovascular disease; *CVH* cardiovascular health; *HF* heart failure; *MI* myocardial infarction

## Discussion

Our main finding was that more time spent with a better CVH score was associated with a lower risk of developing CVD events, MI, HF, stroke, or all-cause mortality in later life, whereas we did not find a significant association of the duration of a better CVH category with AF. In addition, the number of years spent in a better CVH is nonlinearly correlated with all-cause mortality, CVD events, and stroke. The results indicated that more than 6 years spent in a better CVH status was associated with a decreased risk of CVD events or mortality. Overall, our findings support that adults need to maintain healthy lifestyles or behaviors over their whole life course.

Previous studies have reported that ideal CVH status or scores were associated with a decreased risk of CVDs [27, 28] and all-cause mortality [29, 30]. Recently, a systematic review and meta-analysis including 12 prospective cohort studies showed that meeting 5 to 7 ideal CVH metrics was associated with the lowest hazard for CVDs (HR: 0.28; 95% CI 0.23–0.33), which offers an important protective effect for CVDs [31]. In addition, based on the two prospective cohort studies also indicated that favorable CVH status was associated with a lower HF risk compared to unfavorable CVH (HR_CKB_: 0.85, 95% CI 0.81–0.90; HR_UKB_: 0.80, 95% CI 0.77–0.82) [32]. To date, several studies have considered the association of the changes in CVH status or scores over time with clinical outcomes at follow-up [16, 33, 34]. Prior studies evaluating the trends of CVH over 20 years (5 CVH trajectories: low-stable, moderate-decreasing, moderate-increasing, high-stable I, and high-stable II) indicated that improvement or attainment of better CVH metrics through midlife to late life was associated with a lower CVD prevalence and better cardiovascular structure and function [33, 34]. Recently, a study during a follow-up of 10 years showed that high-stable groups could be decreased 64–76% risk of MI in reference to the low-stable group [18]. Similarly, studies also demonstrated that the elevated-stable group could reduce the risk of arterial stiffness (HR: 0.23; 95% CI 0.18–0.29) [35], whereas the declining CVH status group was associated with a higher risk of carotid intima-media thickness (HR: 2.40; 95% CI 1.30–4.50) [36]. Our findings are consistent with a protective effect of ideal CVH status on health outcomes. Application of these 7 simple measures would be a patient-centered and cost-effective way of prevention and management of CVDs. For ideal CVH metrics, it is mechanistically plausible that it has a protective effect on CVDs, such as HF, stroke, or MI [32]. For example, factors similar to those associated with incident HF, including obesity, blood pressure, glucose, smoking, and total cholesterol, were associated with a constellation of myocardial phenotypes classically observed before the onset of frank HF (specifically with preserved ejection fraction), such as left ventricular hypertrophy or concentric LV remodelling [37, 38]. These potential mechanisms include increases in atherogenic lipids, cardiac preload and afterload, and neurohormonal disruption [37, 39]. Our investigation extends prior observations by suggesting that the effects were similar when the duration spent in a better CVH category was modeled. Therefore, prior studies emphasize the importance of promoting or preserving ideal CVH status throughout life to prevent the incidence of CVDs and mortality.

We extended those prior analyses to examine the duration individuals lived in a better CVH and also to consider a range of clinical outcomes, such as incident CVDs and their subtypes, and all-cause mortality. Considering the duration of time lived in a better CVH score category helps us better understand the association of the cumulative burden of lifestyle factors with clinical outcomes. Only two studies reported the association of the duration spent in a special CVH category with clinical outcomes. Data from the Framingham Offspring Study showed that each 5 years spent in ideal or intermediate CVH was associated with a lower risk of incident CVD events (HR: 0.73; 95% CI 0.63–0.85) and all-cause mortality (HR: 0.86; 95% CI 0.76–0.97) over 15 years [19]. In a Korean Genome and Epidemiology Study Ansung-Ansan cohort, individuals who were maintained for more than 10 years had a lower CVD risk (HR: 0.22; 95% CI 0.08–0.60) than those who were maintained for less than 5 years [20]. Similarly, our findings showed that individuals with more than 4 years spent in an ideal or intermediate CVH category had a negative relationship with CVD events (HR: 0.60; 95% CI 0.56–0.63) and mortality (HR: 0.77; 95% CI 0.74–0.81) compared with “** ≤ **4 years” group. These findings suggest that maintenance longer and better CVH status was associated with health outcomes. Modified CVH metrics may directly relate to vascular aging, such as vascular structural remodeling, vascular homeostasis, or atherogenesis [34, 40, 41]. In addition, we observed statistically significant effect modification by age of the associations between duration in a specific CVH category and CVD events or all-cause mortality. We found that the association was most pronounced in younger individuals (younger than age 65) (HR_<65 years_: 0.54, 95% CI 0.50–0.59 vs. HR_≥65 years_: 0.68; 95% CI 0.63–0.73 for CVD events; HR_<65 years_: 0.67, 95% CI 0.60–0.73 vs. HR_≥65 years_: 0.91; 95% CI 0.86–0.96 for all-cause mortality). The older adults (ages 65 and older) for modifying better CVH metrics are limited by time, body condition, longstanding lifestyles, and other reasons. The individual-level CVH score decreased with age and these decreases might start earlier than expected [36, 42]. Numerous studies have found that the decline of CVH score in early life was associated with a later risk of CVDs [42–44]. This suggests that the younger individuals might benefit much from the better chance of CVH than the older.

Although maintaining a better CVH for more than 4 years was associated with a lower CVD or mortality risk, brief maintenance was not meaningful. Previous studies [19, 20] reflected the associations of duration lived in ideal or intermediate CVH with outcomes but did not assume nonlinear relationships. For instance, a cohort study from the Kailuan community-based cohort showed that cumulative CVH (cumCVH) had a significant inverse linear relationship with brachial-ankle pulse wave velocity (*P* < 0.001) and the highest quintile of cumCVH was associated with a 36% significantly lower incidence of arterial stiffness using linear regression model [25]. Another study also presented that every additional year lived with a 1-unit increase in ideal CVH was associated with a 24% significantly lower incidence of diabetes [45]. In contrast to, our results illustrated that the number of years spent in a specific CVH was nonlinearly associated with the development of mortality, CVD events, and stroke. The curves showed that more than 6 years spent in a better CVH status was associated with a decreased risk of CVD events and their subtypes. In addition, we found that the number of years spent in a better CVH associated with the lowest risk of all-cause mortality was 6 years, although the associations were not statistically significant (U shaped). Similarly, among 1445 participants, maintaining a better CVH for 5 years was associated with decreased risk of CVDs (HR: 0.73; 95% CI 0.63–0.85). Prior study reported that individuals maintaining a better CVH for > 10 years had significantly lower risk for CVDs among Korean adults [20]. The subtle differences of these results might be caused by the difference of population, definition of CVH components or each examination cycles year. According to our results, RCS curves showed that as time spent in better CVH increases, the risk of clinical outcomes decreases. Therefore, prolongation of better CVH status is considered essential. However, it can be a challenge for individuals to change their worse CVH status [19] or maintain better CVH status. In light of these findings, public health polices aim to foster healthy lifestyles or behaviors, promote better CVH and prevent diseases in earlier life.

## Strengths and limitations

The strengths of our study include, first, the Kailuan study was the availability of data collected for nearly 13 years from a large community-based population, which allowed us to perform this study with sufficient statistical power. Second, we conducted a new perspective to explore the association of maintaining the time of ideal CVH with outcomes, not just measuring exposure at baseline. To the best of our knowledge, this is the first study to explore the association of the duration maintaining a better CVH with cardiovascular outcomes and all-cause mortality in China. Furthermore, our study also had several limitations. First, physical activity and diet were assessed by a self-report questionnaire, which might lead to an underestimation of the associations for maintaining specific CVH categories. Second, CVH score trajectory analysis was not included in this analysis, and therefore, the fluctuations in CVH scores over time were not evaluated. However, we focus on the duration of maintaining ideal or intermediate CVH rather than CVH itself. Third, due to the small number of participants in ideal CVH, we combined individuals with intermediate and ideal CVH categories. Therefore, the separate effect of ideal CVH on outcomes was not assessed. Finally, although we controlled for a range of potential confounding factors, unmeasured and residual confounding could not be completely avoided.

## Conclusions

In this study, our finding from a large-sample prospective cohort study indicated that longer time lived in intermediate or ideal CVH in midlife might improve longevity and decrease CVDs over the whole life course, and these benefits are more in the younger than in the older adults. These findings suggest the importance of promoting and maintaining healthy lifestyles for cardiovascular health and establishing public health policies that promote people’s health and longevity.

## Supplementary Information

**Additional file 1: Table S1.** The definition of ideal cardiovascular health score.

**Additional file 2: Table S2.** Sensitivity analyses of association between duration of time lived in intermediate or ideal CVH and outcomes.

## Data Availability

Data are available on reasonable request from the corresponding author.

## Abbreviations

aHR: Adjusted hazard ratio
AF: Atrial fibrillation
BMI: Body mass index
BP: Blood pressure
CI: Confidence interval
CVD: Cardiovascular disease
CVH: Cardiovascular health
FBG: Fasting blood glucose
HF: Heart failure
IQR: Interquartile range
MI: Myocardial infarction
RCS: Restricted cubic splines
TC: Total cholesterol
