# Supplementary material for: Time spent in a better cardiovascular health and risk of cardiovascular diseases and mortality: a prospective cohort study
Source: J Transl Med. 2023 Jul 14;21:469. doi: 10.1186/s12967-023-04252-x (PMC10349449; doi:10.1186/s12967-023-04252-x)
Supplement: Supplementary file 1 — Additional file 1: Table S1. The definition of ideal cardiovascular health score. [file 12967_2023_4252_MOESM1_ESM.pdf]

Additional file Tables

Table S1. The definition of ideal cardiovascular health score

Metrics	Poor	Intermediate	Ideal
**Smoking**	Current smoking	Former smoking	Never smoking
**BMI**[a]	≥ 28 kg/m^2	25 to 28 kg/m^2	18 to 24.9 kg/m^2
**Salt preference**	High (> 12g/day)	Medium (6g-12g/day)	Low (< 6g/day)
**Physical activity**	Inactive	Moderately active	Active
**TC**			
**untreated**	≥ 240 mg/dL	200-239 mg/dL	< 200 mg/dL
**treated**	≥ 200 mg/dL	< 200 mg/dL	-
**FBG**			
**untreated**	≥ 7.0 mmol/L	5.6-6.9 mmol/L	< 5.6 mmol/L
**treated**	≥ 5.6 mmol/L	< 5.6 mmol/L	-
**BP**			
**untreated**	SBP ≥ 140 mmHg or DBP ≥ 90 mmHg	SBP: 120-139 mmHg and DBP: 80-89 mmHg	SBP < 120 mmHg or DBP < 80 mmHg
**treated**	SBP > 120 mmHg or DBP > 80 mmHg	SBP < 120 mmHg or DBP < 80 mmHg	-

[a]According to the Chinese-specific criteria, participants were categorized into normal weight (18.5 ≤ BMI < 24.0 kg/m^2), overweight (24.0 ≤ BMI < 28.0 kg/m^2), and obese (BMI ≥ 28.0 kg/m^2).

Abbreviation: BMI = body mass index; BP = blood pressure; DBP = diastolic blood pressure; FBG = fasting blood glucose; SBP = systolic blood pressure; TC = total cholesterol
