# Supplementary material for: Time spent in a better cardiovascular health and risk of cardiovascular diseases and mortality: a prospective cohort study
Source: J Transl Med. 2023 Jul 14;21:469. doi: 10.1186/s12967-023-04252-x (PMC10349449; doi:10.1186/s12967-023-04252-x)
Supplement: Supplementary file 2 — Additional file 2: Table S2. Sensitivity analyses of association between duration of time lived in intermediate or ideal CVH and outcomes. [file 12967_2023_4252_MOESM2_ESM.docx]

**Table S2**. Sensitivity analyses of association between duration of time lived in intermediate or ideal CVH and outcomes

| **Outcomes** | **SHR (95% CI)^a^** | **HR (95% CI)^b^** | **HR (95% CI)^c^** |
| --- | --- | --- | --- |
| **CVD events** | 0.60 (0.56-0.63) | 0.74 (0.69-0.79) | 0.59 (0.56-0.63) |
| **Stroke** | 0.58 (0.54-0.63) | 0.73 (0.67-0.80) | 0.58 (0.54-0.62) |
| **MI** | 0.57 (0.49-0.67) | 0.77 (0.63-0.93) | 0.57 (0.48-0.66) |
| **AF** | 0.84 (0.70-1.01) | 0.94 (0.76-1.18) | 0.86 (0.72-1.04) |
| **HF** | 0.61 (0.54-0.69) | 0.68 (0.59-0.78) | 0.59 (0.52-0.66) |
| **All-cause mortality** | - | 0.83 (0.78-0.88) | 0.75 (0.71-0.78) |

^a^Considering non-CVDs death as a competing risk event rather than a censoring event, Fine-Gray competing risk model was applied to address this issue.

^b^Model was further adjusted CVH score at visit 4, based on the full model.

^c^Time-dependent cox model was conducted.

Abbreviation: AF = atrial fibrillation; CI = confidence interval; CVD = cardiovascular disease; CVH = cardiovascular health; HF = heart failure; HR = hazard ratio; MI = myocardial infarction; SHR = sub-distribution hazard ratio
